# Optimization of Ultrasound-Assisted Microwave Encapsulation of Peanut Oil in Protein-Polysaccharide Complex

**DOI:** 10.17113/ftb.62.01.24.8206

**Published:** 2024-03

**Authors:** Sachin S. Bhuva, Navnit K. Dhamsaniya, Gopal V. Marviya

**Affiliations:** 1 Department of Processing and Food Engineering, CAET, Junagadh Agricultural University, 362001 Junagadh, Gujarat, India; 2 Polytechnic in Agro-Processing, Junagadh Agricultural University, 362001 Junagadh, Gujarat, India; 3 Krishi Vigyan Kendra, Targhadia, Junagadh Agricultural University, 362023 Rajkot, Gujarat, India

**Keywords:** peanut oil, encapsulation, encapsulated peanut oil, microwave drying, corn starch, whey protein isolate

## Abstract

**Research background:**

Peanut oil (*Arachis hypogaea* L.) is a rich source of unsaturated fatty acids. Its consumption has been reported to have biological effects on human health. Unsaturated, especially polyunsaturated fatty acids (PUFA) found in peanut oil are highly susceptible to oxidation, leading to the formation of harmful compounds during processing and storage. The aim of this study is to prevent the oxidation of peanut oil PUFA by encapsulation in a protein-polysaccharide complex using microwave drying.

**Experimental approach:**

The combined effect of corn starch (CS) and whey protein isolate (WPI) was evaluated for ultrasound-assisted microwave encapsulation of peanut oil to prevent oxidative degradation. The effect of independent parameters, *viz*. CS:WPI mass ratio (1:1 to 5:1), lecithin mass fraction (0–5 %), ultrasonication time (0–10 min) and microwave power (150–750 W) on the encapsulation of peanut oil was evaluated using response surface methodology (RSM). The process responses, *viz*. viscosity and stability of the emulsion, encapsulation efficiency, peroxide value, antioxidant activity, free fatty acids (FFA), moisture, angle of repose and flowability (Hausner ratio (HR) and Carr’s Index (CI)) were recorded and analysed to optimize the independent variables.

**Results and conclusions:**

The viscosity of all emulsions prepared for encapsulation by ultrasonication ranged from 0.0069 to 0.0144 Pa·s and more than 90 % of prepared combinations were stable over 7 days. The observed encapsulation efficiency of peanut oil was 21.82–74.25 %. The encapsulation efficiency was significantly affected by the CS:WPI mass ratio and ultrasonication. The peroxide value, antioxidant activity and FFA ranged from 1.789 to 3.723 mg/kg oil, 19.81–72.62 % and 0.042–0.127 %, respectively. Physical properties such as moisture content, angle of repose, HR and CI were 1.94–8.70 %, 46.5–58.3°, 1.117–1.246 and 10.48–22.14 %, respectively. The physical properties were significantly affected by surface properties of the capsules. The higher efficiency (74.25 %) of peanut oil encapsulation was achieved under optimised conditions of CS:WPI mass ratio 1.25, 0.25 % lecithin, 9.99 min ultrasonication and 355.41 W microwave power.

**Novelty and scientific contribution:**

The results of this work contribute to the fields of food science and technology by providing a practical approach to preserving the nutritional quality of peanut oil and improving its stability through encapsulation, thereby promoting its potential health benefits to consumers and applications in various industries such as dairy and bakery.

## INTRODUCTION

Peanut (*Arachis hypogaea* L.), widely known as the king of oilseeds, is an important food source and a significant oilseed crop, containing approx. 48–50 % oil (*1*). Renowned for its pale yellow colour and nutty flavour, peanut oil is highly regarded for its culinary applications, contributing to the texture, taste and offering numerous health benefits, including the reduction of cardiovascular diseases (*2*-*5*). In line with recommendations from the World Health Organization (WHO) and the Food and Agriculture Organization (FAO), Halvorsen and Blomhoff (*6*) reported that the consumption of edible oil enriched with polyunsaturated fatty acids (PUFA) has been advocated to mitigate the risk of cardiovascular diseases. Notably, linoleic acid, a major component of PUFA, plays a vital role in reducing the levels of low-density lipoprotein (LDL) and blood cholesterol.

Peanut oil is abundant in monounsaturated fatty acids (MUFA) and polyunsaturated fatty acids (PUFA), which make up about 80 % of its total fatty acid composition (*7*). In addition, PUFA contributes 17–32 % of peanut oil fatty acids (*8*). A higher percentage of PUFA indicates an increased susceptibility to oxidative rancidity (*9*). Factors such as exposure to oxygen, heat, light and moisture can lead to the breakdown of the double bonds within the fatty acid structure, resulting in rancidity (*10*) and the formation of undesirable products, including peroxides, dienes and ketones.

To combat the oxidative degradation of PUFA-rich oils like peanut oil, the oil is encapsulated within a protective wall matrix (*11*, *12*), which significantly delays oxidation (*13*). The process of oil encapsulation involves two main steps: emulsification and solvent evaporation. The selection of an appropriate wall material for coating is crucial to ensure flowability, stability and shelf-life of the final product (*14*). Whey proteins, when combined with carbohydrates as carrier materials, have shown effective core encapsulation properties. Carbohydrates act as matrix-forming materials, while whey proteins serve as film-forming and emulsifying agents (*15*). In this study, a complex of corn starch and whey protein isolates was chosen as the wall material, with soy lecithin used as a surfactant and binding agent.

Different methods have been used to evaporate the solvent from the emulsion during encapsulation, aiming to extend the shelf-life, control the release and retain bioactive compounds. The most commonly used methods for encapsulation are spray drying (*16*-*18*) and freeze drying (*19*, *20*). However, the high processing temperatures involved in spray drying and the porous structure of the capsules in freeze drying have been observed to adversely affect the stability of the core oil. Consequently, microwave-assisted encapsulation has emerged as a potential alternative to overcome these limitations (*21*, *22*). Microwave-assisted evaporation of the solvent is characterized by internal vapour formation and volumetric heating. The dielectric properties play a crucial role in improving the efficiency of encapsulation, with a lower dielectric constant recommended for the core oil than for the wall materials, enabling the microwave to solidify the wall material shell around the core (*23*). This method significantly saves process time and improves product quality (*24*). Considering the susceptibility of vegetable oils to high temperatures, microwave-assisted encapsulation limits the surface temperature, while the rapid and higher vapour pressure gradient provides mechanical stability to the oil capsules, ensuring product quality and nutritional value (*25*).

While a few studies have focused on microwave-assisted encapsulation of vegetable oils, no attempts have been made to encapsulate peanut oil specifically. Therefore, the present study aims to investigate the optimization of independent parameters for microwave-assisted encapsulation of peanut oil within a protein-polysaccharide complex using corn starch and whey protein isolates as the wall material.

## MATERIALS AND METHODS

### Procurement of materials

Peanut pods of cultivar GJG-32 were procured from the Main Oilseed Research Station of Junagadh Agricultural University (Gujarat, India). The oil was extracted using the wooden ghani (a traditional Indian machine used for extraction of oil from oilseeds) and stored safely in a sealed container at normal atmospheric conditions ((26±2) °C). Food grade corn starch (CS) and lecithin were procured from industrial market of Ahmedabad, India. Whey protein isolate (WPI) was purchased from ProFoods Nutrition Pvt. Ltd. (Mumbai, India). The chemicals used for the evaluation of different properties, as described in different methods, were of analytical grade.

### Emulsion preparation

Distilled water was used as a solvent to prepare a complex of protein and polysaccharide, *i.e.* corn starch and whey protein isolates. The CS:WPI ratio varied from 1:1 to 5:1 (total solids 50 %). The oil phase was prepared by adding *w*(lecithin)=0–5 % to the oil. The oil mass fraction was kept constant at 20 %. The oil phase was blended with aqueous phase containing the above complex to prepare oil-in-water emulsion. Coarse emulsion was sonicated (0–10 min) at 20 kHz using *d*=13 mm probe (amplitude 75 %, pulse rate 40:20, model VC 505; Sonics, Newtown, CT, USA).

### Emulsion properties

The viscosity and stability of the emulsion prepared for encapsulation were measured. The viscosity was measured by Brookfield viscometer (LV DV-II+ Pro; Brookfield Engineering Laboratories, Middleboro, MA, USA) with spindle 61 at 100 rpm (*26*). The emulsion stability was measured by height of separated serum layer (*27*). For this, 10 mL emulsion were poured in 15-mL centrifuge tubes and kept for 7 days at 24 °C. Then, the serum layer height (*h*_s_) against the total height of the emulsion (*h*_e_) was compared. The emulsion stability (%) was calculated using the following equation:


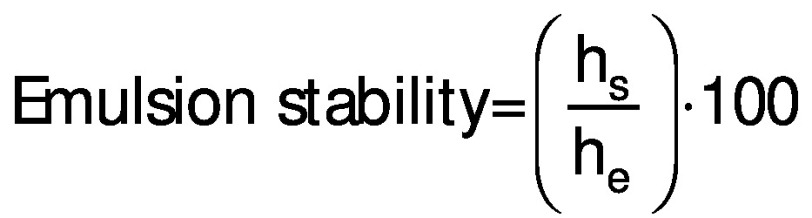
 /1/

### Microwave-assisted encapsulation of peanut oil

The emulsion prepared after ultrasonication was dried in a microwave oven (CE76JD/XTL; Samsung, New Delhi, DL, India) to evaporate the solvent. Different microwave powers (150–750 W at 2450 Hz) were applied for 14 min to the 100-mL samples. A central composite rotatable design (CCRD) of the response surface methodology (RSM) was used to optimize the encapsulation of peanut oil. Five levels of all four factors: CS:WPI mass ratio (X_1_), lecithin mass fraction (X_2_), ultrasonication time (X_3_) and microwave power (X_4_) were chosen to evaluate their effect on the encapsulation efficiency, peroxide value, antioxidant activity, moisture content and Hausner ratio (Table 1), obtaining a total of 30 treatment combinations, including six centre points. The following polynomial (quadratic) equation was fitted to analyse the data:


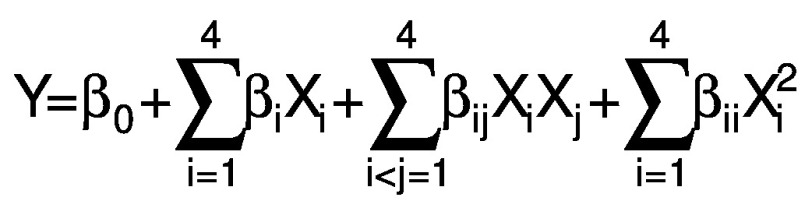
 /2/

**Table 1:** Experimental design with real and coded values of independent variables

Independent variable		Coded value				
-2	-1	0	+1	+2
*m*(CS):*m*(WPI)	(X_1_)	1	2	3	4	5
*w*(lecithin)/%	(X_2_)	0	1.25	2.5	3.75	5
*t*(ultrasonication)/min	(X_3_)	0	2.5	5	7.5	10
*P*(microwave)/W	(X_4_)	150	300	450	600	750

CS=corn starch, WPI=whey protein isolate

where Y represents response variable, X_i_ and X_j_ are the independent variables that affect the response, and β_0_, β_i_, β_ij_ and β_ii_ are regression coefficients for intercept, linear, interaction and quadratic terms, respectively.

### Encapsulation efficiency

Encapsulation efficiency was calculated from the surface oil and total oil content (*16*):


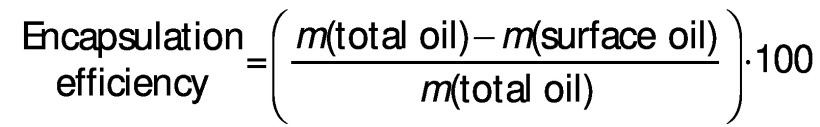
 /3/

Surface oil was measured by adding samples (3 g) in 50 mL of hexane and vortexing for 30 s. The mixture was filtered through a Whatman No. 1 filter paper and the capsules were rinsed twice with 20 mL hexane. The filtered solution was left on a hot plate at 80 °C to evaporate the solvent and weighed to determine the surface oil. The total mass of oil was presumed to be equal to the initial mass of oil.

### Measurement of chemical properties of encapsulated peanut oil

#### Peroxide value

Peroxide value was measured as an indicator of lipid oxidation. A sample (1 g) was extracted in 10 mL distilled water (*17*). A volume of 400 μL of extract was vortexed for 10 s after adding 1.5 mL of *V*(isooctane):*V*(isopropanol)=2:1.To measure the peroxide value, the supernatant was taken from the separate phases. Spectrophotometer (GENESYS 50; ThermoFisher Scientific, Mumbai, MH, India) was used to measure peroxide value according to the International Dairy Federation (IDF) standard method with minor modifications (*28*). A volume of 400 μL of extract was added to 9.6 mL of a *V*(chloroform):*V*(methanol)=7:3 mixture. Colour was developed with 50 μL of ammonium thiocyanate and iron(II) chloride solution which was freshly prepared by adding 3.94 M thiocyanate to Fe(II) solution. The samples were briefly vortexed and allowed to react in the dark for 30 min. The absorbance was measured at 500 nm and the peroxide value (mg per kg oil) was calculated according to the following equation:


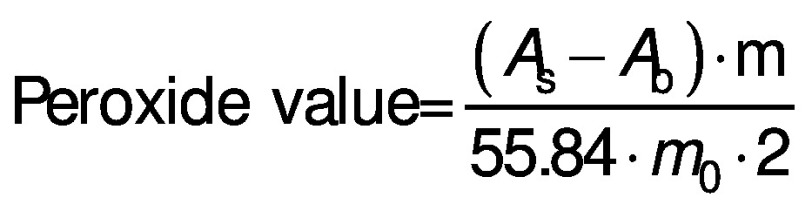
 /4/

where *A*_s_ is the absorbance of the sample, *A*_b_ is the absorbance of blank, m is the slope of standard curve and *m*_0_ is the mass in grams of the sample. Standard was prepared with iron(III) chloride.

#### Antioxidant activity

The antioxidant activity was measured by the DPPH free radical scavenging assay with minor modifications (*29*). After alcoholic extraction (1:10), the alcohol was added to different concentrations of samples to make a total volume of 2 mL. Fresh DPPH solution (0.012 g per 100 mL alcohol) was added to the samples and shaken vigorously for thorough mixing. The entire process was conducted in complete darkness and the samples were allowed to react for 30 min. The absorbance was measured at 517 nm and the antioxidant activity (%) was calculated using the following equation:


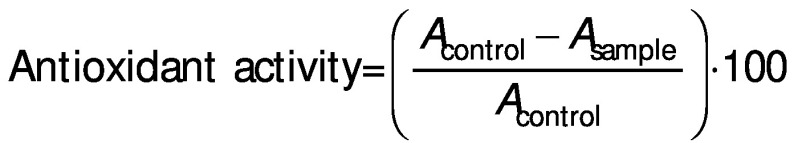
 /5/

#### Free fatty acids

The content of free fatty acids was measured by AOAC Official Method 922.11 (*30*). The oil from the sample was extracted using Soxhlet extraction unit and 1 g of oil was taken. Then, 25 mL of diethyl ether and 25 mL of alcohol were added. The samples were then titrated against 0.5 M NaOH with phenolphthalein indicator until a pink colour developed and persisted for at least 15 s.

### Measurement of physical properties

Moisture content, angle of repose and flowability are important physical properties of encapsulated oil during transportation and storage. They are affected by the process variables temperature and time, composition of the materials and also by the efficiency of encapsulation.

#### Moisture content

The moisture content of the samples was determined in a hot air oven (*31*). A mass of 2 g of sample was placed in the hot air oven at 105 °C until constant mass was achieved. Moisture was calculated as mass fraction in % of water removed from the sample on wet basis.

#### Angle of repose

The angle of repose is the angle (°) formed relative to the horizontal plane on which material is piled. It was measured by passing a known preweighed sample gravimetrically on a circular platform (*25*) and calculated as:


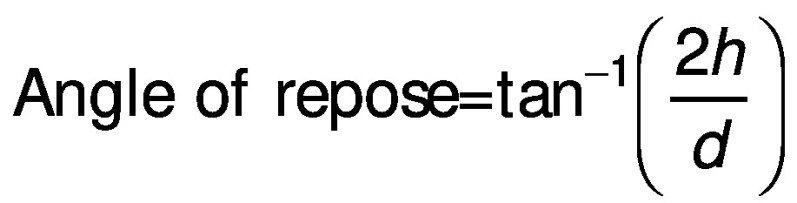
 /6/

where *h* is the height of the heap and *d* is the diameter of the platform.

#### Flowability

Flowability is assessed by Hausner ratio (HR) and Carr’s index (CI). The Hausner ratio is an indication of the compressibility of the powdery material (*32*). The Carr’s index, also known as compressibility index (C), is another indicator of flowability. The bulk density (*ρ*_b_) of the capsules was calculated according to Mishra *et al*. (*33*). A mass of 5 g of sample was poured in 50-mL measuring cylinder. Tapped density (*ρ*_t_) was measured after tapping the bench 50 times by hand from the height of 10 cm. The formulae for HR and CI (%) are as follows:


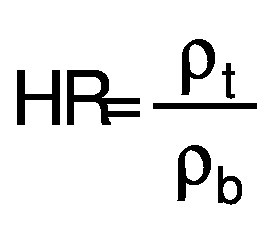
 /7/

and


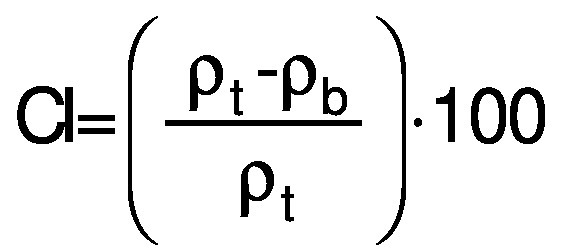
 /8/

### Statistical analysis

An analysis of variance (ANOVA) was conducted to observe significant differences among the independent variables (p<0.05). The optimal treatment conditions were selected using the desirability function. Regression coefficients were determined and three-dimensional graphs were generated using Design Expert v. 11 statistical software (*34*). The correlation analysis, assessing the statistical association by measuring the strength and direction of the relationship between two variables, was conducted for the responses.

## RESULTS AND DISCUSSION

### Viscosity and stability of emulsion

The viscosity of the emulsion plays a crucial role in determining its flow behaviour, heat and mass transfer, and aeration properties. The viscosity of all the emulsions prepared by ultrasonication ranged from 0.0069 to 0.0144 Pa·s. Higher amounts of corn starch resulted in lower viscosity. The adhesive properties of the protein-polysaccharide complex affected the emulsion viscosity, with lecithin, a binding agent, contributing to an increase in viscosity. Carneiro *et al*. (*18*) also reported an increase in the emulsion viscosity with the use of gum Arabic and modified starch.

Emulsion stability refers to the tendency of the emulsion to separate into two or more phases, which is influenced by coalescence. The samples stored at room temperature (24 °C) showed highly fluctuating stability. The lowest stability (34.52 %) was observed in samples with higher corn starch and lower lecithin content, indicating insufficient emulsifying agents to bind the oil phase to the wall matrix. However, most samples showed more than 90 % stability over 7 days. The combination of protein and lecithin acted as an emulsifying agent, enhancing the binding of the oil and providing higher emulsion stability. Benichou *et al*. (*35*) reported that the protein-polysaccharide complex improved functional properties, including emulsion stability. The presence of whey protein caused flocculation, leading to the development of a network structure that prevents separation (*36*).

### Efficiency of peanut oil encapsulation

The encapsulation efficiency was determined to evaluate the effect of independent parameters on the encapsulation process. The encapsulation efficiency ranged from 21.82 to 74.25 % (Table 2).

**Table 2:** Encapsulation efficiency and chemical parameters of encapsulated peanut oil determined in 30 tests of the experiment

Test	X_1_	X_2_	X_3_	X_4_	EE/%	PV/(mg/kg)	AA/%	FFA/%
1	3	5	5	450	51.34	3.514	46.60	0.111
2	2	1.25	7.5	300	54.06	2.418	65.20	0.113
3	3	2.5	5	450	41.83	3.104	48.71	0.099
4	1	2.5	5	450	46.54	3.127	49.17	0.127
5	4	3.75	7.5	300	34.77	2.449	43.72	0.042
6	3	2.5	5	450	41.03	3.171	38.73	0.099
7	3	0	5	450	54.31	3.723	34.64	0.085
8	2	1.25	7.5	600	59.35	3.156	36.61	0.071
9	4	1.25	2.5	300	33.32	3.420	72.62	0.099
10	2	1.25	2.5	300	54.54	3.530	60.95	0.099
11	2	1.25	2.5	600	74.25	3.040	19.82	0.092
12	3	2.5	5	450	47.33	2.579	39.40	0.085
13	4	3.75	2.5	300	65.29	2.111	68.53	0.085
14	3	2.5	10	450	35.75	2.273	58.55	0.099
15	3	2.5	5	450	50.30	2.511	55.66	0.085
16	4	1.25	2.5	600	44.19	2.740	53.25	0.099
17	2	3.75	2.5	600	32.27	3.095	49.32	0.105
18	5	2.5	5	450	33.37	2.144	38.73	0.099
19	3	2.5	5	150	50.12	2.031	70.06	0.103
20	4	1.25	7.5	300	37.71	1.789	66.11	0.093
21	2	3.75	2.5	300	61.72	3.214	62.93	0.085
22	2	3.75	7.5	300	37.18	2.385	65.81	0.113
23	4	3.75	2.5	600	44.80	3.185	62.78	0.110
24	3	2.5	5	450	41.75	2.853	56.91	0.081
25	4	3.75	7.5	600	21.82	2.692	35.40	0.113
26	3	2.5	0	450	43.36	3.304	51.32	0.099
27	3	2.5	5	750	35.11	2.914	62.36	0.113
28	3	2.5	5	450	51.89	2.569	43.42	0.085
29	2	3.75	7.5	600	37.48	2.956	50.23	0.127
30	4	1.25	7.5	600	47.74	1.982	43.72	0.071

X_1_, X_2_, X_3_, X_4_ represent experimental variables: *m*(CS):*m*(WPI), *w*(lecithin)/%, *t*(ultrasonication) and *P*(microwave), respectively. EE=encapsulation efficiency, PV=peroxide value, AA=antioxidant activity, FFA=free fatty acids, CS=corn starch, WPI=whey protein isolate

**Fig. 1:**
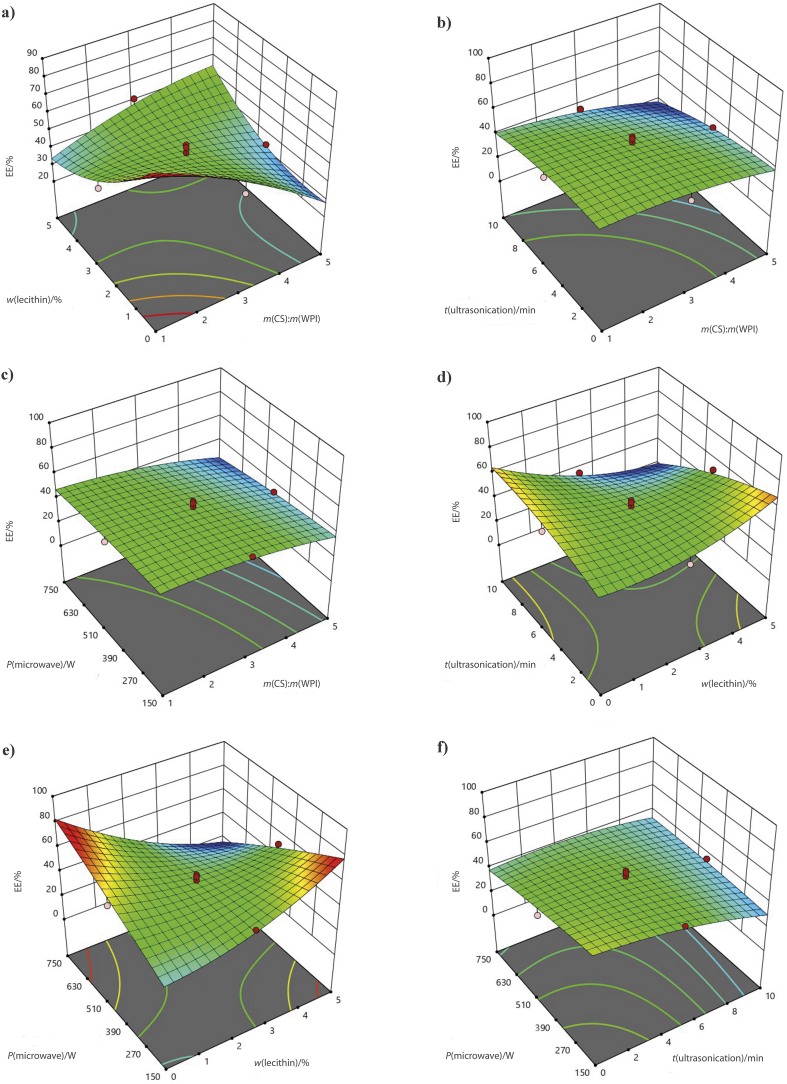
The effect of the interaction of independent parameters: a) *m*(CS):*m*(WPI) and *w*(lecithin), b) *m*(CS):*m*(WPI) and *t*(ultrasonication), c) *m*(CS):*m*(WPI) and *P*(microwave), d) *w*(lecithin) and *t*(ultrasonication), e) *w*(lecithin) and *P*(microwave), and f) *t*(ultrasonication) and *P*(microwave) on the encapsulation efficiency

The 3D graphs in Fig. 1 show the effect of these parameters on the encapsulation efficiency. Higher protein content in CS:WPI mass ratio for the wall material increased the encapsulation efficiency. The combination of lecithin with protein helped further to increase the efficiency of oil encapsulation. Ultrasonication affected negatively the encapsulation efficiency. A decreasing efficiency of peanut oil encapsulation was observed when lecithin and ultrasonication time were increased.

The results of the analysis of variance (ANOVA) in Table 3 indicated that the CS:WPI mass ratio and ultrasonication time had a significant effect on peanut oil encapsulation. In addition, the interaction of lecithin with all parameters was observed as significant. It interacted positively with CS:WPI mass ratio, while negatively with ultrasonication time and microwave power.

**Table 3:** Analysis of variance (ANOVA) of the quadratic model for encapsulation efficiency

Source	Sum of squares	df	Mean square	F-value	p-value	Remark
Model	2841.80	14	202.99	3.85	0.0070	S
A=*m*(CS):*m*(WPI)	481.77	1	481.77	9.14	0.0086	
B=*w*(lecithin)/%	239.22	1	239.22	4.54	0.0501	
C=*t*(ultrasonication)/min	379.87	1	379.87	7.21	0.0170	
D=*P*(microwave)/W	90.89	1	90.89	1.72	0.2089	
AB	373.30	1	373.30	7.08	0.0178	
AC	7.36	1	7.36	0.1395	0.7140	
AD	4.41	1	4.41	0.0836	0.7764	
BC	267.21	1	267.21	5.07	0.0398	
BD	735.69	1	735.69	13.96	0.0020	
CD	30.38	1	30.38	0.5764	0.4595	
A^2^	27.99	1	27.99	0.5309	0.4774	
B^2^	133.74	1	133.74	2.54	0.1321	
C^2^	33.77	1	33.77	0.6406	0.4360	
D^2^	3.27	1	3.27	0.0620	0.8068	
Residual	790.72	15	52.71			
Lack of fit	676.10	10	67.61	2.95	0.1221	NS
Pure error	114.62	5	22.92			
Cor total	3632.52	29				

A, B, C, D are experimental variables, df=degree of freedom, S=significant, NS=not significant

Higher protein content promotes greater stability and the formation of a shell during sonication, resulting in higher encapsulation efficiency (*37*). Longer ultrasonication time unfolded and aggregated polysaccharides, which resulted in a lower encapsulation efficiency. The incorporation of saccharides into skimmed milk powder has been shown to increase the encapsulation efficiency of fish oil, which is further improved by the use of a surfactant (*38*). It should be noted that comparing results from different studies may vary due to variations in wall materials and processing conditions used by previous researchers. For example, de Paz *et al*. (*39*) reported lower encapsulation efficiency (not exceeding 58.7 %) of encapsulation of β-carotene in liposomes using soy lecithin, while Melgosa *et al*. (*40*) achieved an encapsulation efficiency of around 100 % for encapsulating fish oil in modified starch. Piornos *et al*. (*41*) also reported encapsulation efficiencies ranging from 2.14 to 93.16 % for linseed oil using different concentrations of wall materials.

### Chemical properties

#### Peroxide value of encapsulated peanut oil

The peroxides in the capsules indicate the oxidative degradation. The peroxide mass fraction varied from 1.789 to 3.723 mg/kg (Table 2). It was observed that the composition of the wall material strongly affected the peroxide mass fraction of the capsules. Lower peroxide values were found in the wall material with higher protein content. Ultrasonication significantly decreased the peroxide value. Lower peroxide values were observed with longer ultrasonication times during the preparation of emulsion for encapsulation. The higher amount of whey protein in the complex CS-WPI strengthened protein-protein interactions, as protein molecules unfolded at the droplet surface, leading to flocculation and higher efficiency. The cavitation process of ultrasonication generated impulsive energy, favouring the formation of protein complexes and resulting in better encapsulation and lower peroxide formation.

The presence of whey protein as a protein source in the wall material complex resulted in higher oil oxidation stability (*18*). Higher power generated more energy during high intensity processing, leading to the unfolding of bonds at the molecular level and higher peroxide formation during oil processing (*42*, *43*).

#### Antioxidant activity of encapsulated peanut oil

The antioxidant activity was assessed based on the percentage inhibition, which was found to be influenced by the microwave power. The antioxidant activity of encapsulated peanut oil ranged from 19.82 to 72.62 % (Table 2). The highest activity (72.62 %) was observed with shorter ultrasonication time and lower microwave power. Microwave power is the only significant parameter for antioxidant activity. The effect of the interaction of CS:WPI mass ratio with lecithin mass fraction and ultrasonication time was significant. The increased power in microwave processing resulted in the absorption of energy by the antioxidants, which increased the temperature of the product. This higher temperature promoted oxidation and the loss of volatile compounds, resulting in a slight decrease in inhibition. Additionally, a longer ultrasonication time also contributed to an increase in the emulsion temperature, leading to a slight decrease in the inhibition. Ahmad *et al*. (*44*) reported a negative effect of higher microwave power on antioxidant activity. The findings are also supported by a study on microwave-assisted encapsulation of PUFA-rich vegetable oil investigated by Pattnaik and Mishra (*25*).

#### Free fatty acids of encapsulated peanut oil

Free fatty acids (FFA) indicate the degradation of oil, as they are formed through the hydrolysis of oil into free fatty acids and glycerol due to the splitting of ester bonds. The FFA content (% oleic acid) of peanut oil capsules ranged from 0.042 to 0.127 % (Table 2). The Codex Alimentarius Commission (CAC) set a limit of 0.3 % oleic acid for refined oil (*45*), which corresponds to mass fraction of KOH 0.6 mg/g of oil after conversion with a factor of 1.99. All the samples of encapsulated peanut oil had FFA content within the permissible limit (<0.3 %). An increase in the CS:WPI mass ratio resulted in a lower amount of FFA. The composition of the wall material affected the properties of the developed microstructure. Koc *et al*. (*46*) also reported a decrease in the FFA content with an increased amount of maltodextrin (MD) in the MD:WPI wall material complex. Therefore, the present findings are in agreement with those results.

### Physical properties

#### Moisture content of peanut oil capsules

Moisture content plays a significant role in food processing. Table 4 shows that the moisture content of the peanut oil capsules ranged from 1.94 to 8.70 %. The wall materials, lecithin mass fraction and ultrasonication time were found to have a significant effect on moisture content. The moisture content of capsules increased with an increase in the starch amount. It was also increased with an increase in lecithin mass fraction. Longer ultrasonication decreased the moisture of peanut oil capsules. The effect of interaction of lecithin mass fraction with CS:WPI mass ratio and microwave power was also found significant. Higher moisture mass fractions increase the chances of oxidation and affect the flowability of powdery material (*47*). The higher protein content in the complex acts as a water-binding agent through cross-linking, which resulted in lower moisture mass fraction. The interaction between amounts of protein and lecithin further enhanced water-binding properties, resulting in lower product moisture. A longer duration of ultrasonication allows for more significant interaction with moisture by decreasing size of the particles which create a larger surface area. In addition, a higher microwave power increases the temperature of the product, which reduces the moisture content. The moisture mass fraction was found to be lower at the microwave power of 550 W than at 330 W during the encapsulation of purple sweet potato extract (*22*). A similar trend was also observed by Pattnaik and Mishra (*25*).

**Table 4:** Physical parameters of encapsulated peanut oil determined in 30 tests of the experiment

Test	X_1_	X_2_	X_3_	X_4_	*w*(moisture)/%	AR/°	HR	CI/%
1	3	5	5	450	7.06	49.3	1.117	10.48
2	2	1.25	7.5	300	3.85	46.8	1.174	14.79
3	3	2.5	5	450	3.42	50.6	1.197	18.84
4	1	2.5	5	450	3.49	51.8	1.160	14.78
5	4	3.75	7.5	300	2.46	47.5	1.184	16.00
6	3	2.5	5	450	3.37	48.6	1.238	15.45
7	3	0	5	450	3.81	48.8	1.141	14.14
8	2	1.25	7.5	600	1.99	46.5	1.179	15.20
9	4	1.25	2.5	300	6.61	48.6	1.177	13.71
10	2	1.25	2.5	300	4.65	49.3	1.219	17.99
11	2	1.25	2.5	600	1.94	54.0	1.236	17.66
12	3	2.5	5	450	4.05	51.2	1.246	15.32
13	4	3.75	2.5	300	4.16	46.5	1.226	14.95
14	3	2.5	10	450	2.46	53.5	1.170	14.50
15	3	2.5	5	450	3.46	50.6	1.210	17.35
16	4	1.25	2.5	600	7.12	52.4	1.183	16.47
17	2	3.75	2.5	600	8.70	49.5	1.203	16.90
18	5	2.5	5	450	6.96	50.6	1.206	18.64
19	3	2.5	5	150	6.79	48.9	1.205	16.98
20	4	1.25	7.5	300	8.34	49.3	1.129	12.40
21	2	3.75	2.5	300	7.27	52.9	1.240	19.36
22	2	3.75	7.5	300	6.03	50.6	1.170	14.56
23	4	3.75	2.5	600	6.28	54.0	1.231	18.79
24	3	2.5	5	450	4.50	49.9	1.199	16.57
25	4	3.75	7.5	600	5.42	53.5	1.148	12.44
26	3	2.5	0	450	6.93	58.3	1.235	22.14
27	3	2.5	5	750	3.88	52.9	1.182	15.43
28	3	2.5	5	450	4.69	52.0	1.202	17.74
29	2	3.75	7.5	600	6.99	51.8	1.153	13.58
30	4	1.25	7.5	600	6.69	48.9	1.196	16.38

X_1_, X_2_, X_3_, X_4_ represent experimental variables: *m*(CS):*m*(WPI), *w*(lecithin)/%, *t*(ultrasonication) and *P*(microwave), respectively. AR=angle of repose, HR=Hausner ratio, CI=Carr’s index, CS=corn starch, WPI=whey protein isolate

#### Angle of repose of peanut oil capsules

The angle of repose of the peanut oil capsules varied from 46.5 to 58.3° with a 20 % oil load (Table 4). It was significantly affected by ultrasonication time. Longer ultrasonication decreased the angle of repose of encapsulated peanut oil. A higher ultrasonication time leads to the formation of uniform globules of smaller size, which are less cohesive. The fine structure of small globules leads to a lower angle of repose. Smaller particles are also more susceptible to higher Van der Waals forces due to their larger surface area. Thus, ultrasonication time had an inverse effect on the angle of repose. However, other factors also contribute to the angle of repose. Microwave power had a direct effect on the angle due to the rough surface of the particles that developed because of lower moisture content. Lecithin amount also contributed to a higher angle of repose due to the change in surface binding properties. Higher moisture content also increased the angle of repose due to water plasticization (*48*). These results are in agreement with those reported by Pattnaik and Mishra (*25*).

#### Flowability of peanut oil capsules

The flowability of the peanut oil capsules was assessed using the Hausner ratio (HR) and Carr's index (CI), which indicate the compressibility of the powdery material. Flowability depends on the surface characteristics of the particles. The HR and CI ranged from 1.117 to 1.246 % and from 10.48 to 22.14 %, respectively (Table 4). A similar range of HR and CI was reported by Pattnaik and Mishra (*25*) for the microwave-assisted encapsulation of vegetable oil. Lower values of HR and CI signify higher flowability. A CI value less than 15 % indicates excellent flowability, while a CI of 25 % or greater reflects poor flowability (*49*). An HR lower than 1.18 indicates good compressibility and higher than 1.35 indicates poor compressibility. The powdery particles produced in this study showed good flowability. The ultrasonication time had a significantly positive effect on flowability by creating smaller particles due to longer duration of vibration. Higher encapsulation efficiency indicates a lower surface oil content, which enhances the flow characteristics of the particles. Gulzar *et al*. (*50*) depicted higher flowability for shrimp oil encapsulation using ultrasonication.

### Correlation and analysis of variance of response parameters

The correlation analysis showed relationships between the response parameters, ranging from +1 to -1, which indicated positive and negative correlations, respectively (Table 5). Encapsulation efficiency was positively correlated with the peroxide value and the flowability (HR and CI) of the peanut oil capsules. Conversely, moisture content and antioxidant activity showed a negative correlation with encapsulation efficiency. These correlations suggested that higher efficiency was associated with lower moisture content and improved flowability of the peanut oil capsules. Additionally, higher encapsulation efficiency corresponded to higher peroxide values and lower antioxidant activity. The angle of repose exhibited a strong positive correlation with Carr's index (CI), indicating reduced flowability at higher angles of repose. Based on these observations, an analysis of variance (ANOVA) was conducted using a quadratic model and the regression coefficients for the response variables are given in Table 6 (p<0.05).

**Table 5:** Correlation between the responses evaluated for encapsulation of peanut oil

	EE	PV	AA	FFA	MC	AR	HR	CI
EE	1.000							
PV	0.149	1.000						
AA	-0.129	-0.221	1.000					
FFA	-0.251	0.198	0.238	1.000				
MC	-0.324	-0.093	0.363	0.313	1.000			
AR	-0.149	0.191	-0.137	0.339	0.154	1.000		
HR	0.355	0.024	-0.044	-0.266	-0.138	0.232	1.000	
CI	0.221	0.092	-0.025	-0.211	0.013	0.438	0.774	1.000

EE=encapsulation efficiency, PV=peroxide value, AA=antioxidant activity, FFA=free fatty acids, MC=moisture content, AR=angle of repose, HR=Hausner ratio, CI=Carr’s index

**Table 6:** Estimated regression coefficients of the quadratic model for responses

Regression coefficient	Response							
EE	PV	AA	FFA	MC	AR	HR	CI
β_0_ (intercept)	45.69	2.80	47.14	0.09	3.92	50.49	1.22	16.88
Linear								
β_1_	-4.48**	-0.22**	0.60	-0.01*	0.52*	-0.13	0.00	-0.05
β_2_	-3.16	-0.02	1.85	0.00	0.53*	0.48	0.00	-0.22
β_3_	-3.98*	-0.27**	-1.21	0.00	-0.58**	-0.92*	-0.02***	-1.49***
β_4_	-1.95	0.14	-7.09***	0.00	-0.17	1.13*	0.00	0.02
Interaction								
β_12_	4.83*	0.06	-4.44*	0.00	-1.69***	-0.37	0.01	0.28
β_13_	-0.68	-0.04	-5.82*	-0.01*	0.15	0.49	0.00	0.44
β_14_	-0.52	0.01	2.69	0.01	0.38	0.92	0.00	0.65
β_23_	-4.09*	0.14	-3.34	0.00	-0.38	0.82	-0.01	-0.40
β_24_	-6.78**	0.13	4.26	0.01***	0.82**	0.21	-0.01	-0.62
β_34_	1.38	0.12	0.31	0.00	-0.06	-0.38	0.00	-0.25
Quadratic								
β_11_	-1.01	-0.06	-0.44	0.00	0.39	-0.10	-0.01	-0.07
β_22_	2.21	0.19*	-1.27	0.00	0.44*	-0.63	-0.02***	-1.17**
β_33_	-1.11	-0.02	2.30	0.00	0.25	1.09*	0.00	0.33
β_44_	-0.35	-0.10	5.12**	0.00	0.41	-0.16	0.00	-0.20

β_0_, β_i_, β_ij_, and β_ii_ are the regression coefficients for intercept, linear, interaction and quadratic terms, respectively. *, ** and ***=significant at p<0.05, p<0.01 and p<0.001, respectively. EE=encapsulation efficiency, PV=peroxide value, AA=antioxidant activity, FFA=free fatty acids, MC=moisture content, AR=angle of repose, HR=Hausner ratio, CI=Carr’s index

### Optimization of peanut oil encapsulation

The effect of CS:WPI mass ratio, lecithin mass fraction, ultrasonication time and microwave power was analysed for the responses encapsulation efficiency (EE), peroxide value (PV), antioxidant activity (AA), moisture (MC) and Hausner ratio (HR). The values of EE, PV, AA, MC and HR varied from 21.82 to 74.25 %, 1.789 to 3.723 mg/kg, 19.81 to 72.62 %, 1.94 to 8.70 % and 1.117 to 1.246, respectively, showing significance of the change in independent variables. The regression equations reflecting the relation between the independent variables and responses (p<0.05) are presented, omitting non-significant terms:


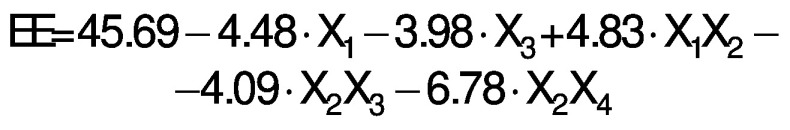
 /9/


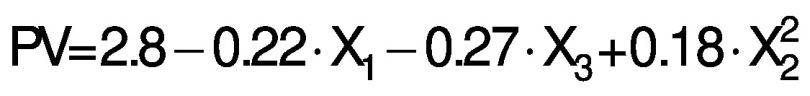
 /10/


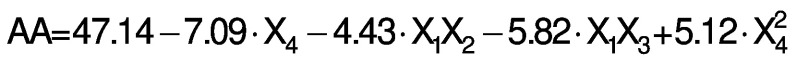
 /11/


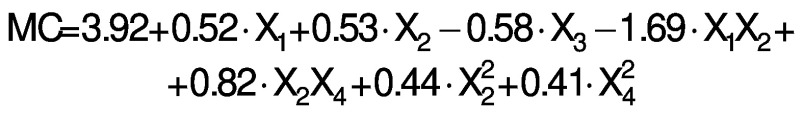
 /12/


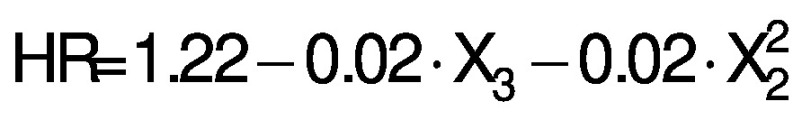
 /13/

Numerical optimization was performed to determine the optimal combination of independent parameters. The criteria were set to maximize EE and AA, and minimize PV, MC and HR. The optimum condition was found to be a CS:WPI mass ratio of 1.25, lecithin mass fraction of 0.25 %, 10 min of ultrasonication and 355.41 W of microwave power. The optimised conditions resulted in an EE of 74.25 %, PV of 2.68 mg/kg, AA of 76.42 %, MC of 1.73 %, HR of 1.117 and a desirability of 0.875.

## CONCLUSIONS

It has been observed that the wall material composition (the mass ratio of corn starch (CS) to whey protein isolate (WPI)) and ultrasonication significantly affected the properties of the peanut oil capsule. The interaction between lecithin and protein during prolonged ultrasonication had a significant effect on the encapsulation efficiency. The microwave power also had a significant effect on the antioxidant activity. The flowability of the prepared peanut oil capsules was found to be acceptable. The content of free fatty acids was within permissible limits after encapsulation. It is concluded that the encapsulation efficiency of peanut oil of 74.25 % can be achieved using CS:WPI mass ratio of 1.25, lecithin mass fraction of 0.25 %, ultrasonication time of 10 min, and microwave power of 355.41 W that results in higher antioxidant activity and flowability, lower moisture, free fatty acids and peroxide value. Thus, encapsulation preserves the quality and provides better stability of the peanut oil. The study successfully demonstrated the encapsulation of peanut oil in a protein-polysaccharide complex using the microwave-assisted technique. Overall, the optimized conditions identified in this study represent a novel encapsulation approach added to the scientific literature for further research and industrial applications to develop encapsulated oil with improved functional and nutritional properties.
